# Molecular characterization and identification of predictors of disease outcome in Saudi colorectal carcinoma

**DOI:** 10.1186/1471-2164-15-S2-O6

**Published:** 2014-04-02

**Authors:** Abdelbaset Buhmeida, Ashraf Dallol, Jaudah Al-Maghrabi, Mahmoud Al-Ahwal, Abdulrahman Sibiany, Mohmmad Al-Qahtani

**Affiliations:** 1 Center of Excellence in Genomic Medicine Research, King Abdulaziz University, P.O. Box: 80216 Jeddah 21586, Kingdom of Saudi Arabia; 2 Department of Pathology, Faculty of Medicine, King Abdulaziz University, Jeddah, Kingdom of Saudi Arabia; 3 Colerectal Cancer Scientific Chair, King Abdulaziz University, Jeddah, Kingdom of Saudi Arabia; 4 Department of Surgery, Faculty of Medicine, King Abdulaziz University, Jeddah, Kingdom of Saudi Arabia

## 

Colorectal Carcinoma (CRC) is a heterogeneous disease with different molecular characteristics associated with the sites from which, the tumours originate. Such heterogeneity is compounded by the multitude of genetic and epigenetic variations acting as passengers or drivers of the tumour. Majority of CRC develops via chromosomal instability (CIN) pathway. CIN is often exacerbated by inactivation of the Wnt signalling pathway "master regulator" APC gene, activating mutations of KRAS or BRAF oncogenes, or deletions of the 18q, and 17p chromosomal regions with deleterious effects on the tumour suppressor genes TP53 and DCC. Defective Mismatch Repair (MMR) pathway results in a subtler form of genomic instability, namely Microsatellite Instability (MSI). High levels of MSI (or MSI-H) in sporadic CRC are usually caused by hypermethylation of the MLH1 promoter. In terms of methylation, the CpG island methylator phenotype (CIMP) pathway is the second most common pathway in sporadic CRC. CIMP-positive (CIMPp) CRC tumours are usually associated with the proximal colon of older females. CIMPp CRC tumours have better prognosis if the tumours are also MSI-H. However, CIMPp CRC tumours that are Microsatellite Stable (MSS) have poor clinical outcome. To gain insight into the molecular mechanisms underpinning CRC in Saudi Arabian patients, we profiled the DNA methylation frequency of key genes (MLH1, MSH2, RASSF1A, SLIT2, HIC1, MGMT, SFRP1, MYOD1, APC, CDKN2A, and other five CIMP markers) in 120 sporadic CRC cases. CRC tumours originating from the rectum, left, and right colons are represented in this cohort. Expression patterns of different proteins playing important role in CRC carcinogenesis also studied by using Immunohistochemistry (IHC) technique and their impact as CRC prognosticators was evaluated.

